# Surgical Debridement Is Superior to Sole Antibiotic Therapy in a Novel Murine Posttraumatic Osteomyelitis Model

**DOI:** 10.1371/journal.pone.0149389

**Published:** 2016-02-12

**Authors:** Johannes Maximilian Wagner, Hannah Zöllner, Christoph Wallner, Britta Ismer, Jessica Schira, Stephanie Abraham, Kamran Harati, Marcus Lehnhardt, Björn Behr

**Affiliations:** Department of Plastic Surgery, BG Bergmannsheil Bochum, Ruhr-University of Bochum, Bochum, Germany; Purdue University, UNITED STATES

## Abstract

**Introduction:**

Bone infections after trauma, *i*.*e*. posttraumatic osteomyelitis, pose one of the biggest problems of orthopedic surgery. Even after sufficient clinical therapy including vast debridement of infected bone and antibiotic treatment, regeneration of postinfectious bone seems to be restricted. One explanation includes the large sized defects resulting from sufficient debridement. Furthermore, it remains unclear if inflammatory processes after bone infection do affect bone regeneration. For continuing studies in this field, an animal model is needed where bone regeneration after sufficient treatment can be studied in detail.

**Methods:**

For this purpose we created a stable infection in murine tibiae by *Staphylococcus aureus* inoculation. Thereafter, osteomyelitic bones were debrided thoroughly and animals were subsequently treated with antibiotics. Controls included debrided, non-infected, as well as infected animals exclusively treated with antibiotics. To verify sufficient treatment of infected bone, different assessments detecting *S*. *aureus* were utilized: agar plates, histology and RT-qPCR.

**Results:**

All three detection methods revealed massive reduction or eradication of *S*. *aureus* within debrided bones 1 and 2 weeks postoperatively, whereas sole antibiotic therapy could not provide sufficient treatment of osteomyelitic bones. Debrided, previously infected bones showed significantly decreased bone formation, compared to debrided, non-infected controls.

**Discussion:**

Thus, the animal model presented herein provides a reliable and fascinating tool to study posttraumatic osteomyelitis for clinical therapies.

## Introduction

One of the biggest problems in the field of orthopedic surgery is infection of bone and soft tissue. Especially posttraumatic osteomyelitis, which is mostly a cause of open fractures, leads to progressive inflammation and bone destruction [1]. Osteomyelitis can be subdivided into hematogenous or posttraumatic spread of bacteria, whereas the incidence of posttraumatic osteomyelitis is 80% [2]. Bone infection after trauma is one of the most challenging problems of orthopedic surgery, whereupon incidence after elective surgery is about 1–5%. However incidence increases to values between 3 and 50% after trauma, depending on the severity [2].

The treatment is often a challenging problem, since germs with variable drug sensitivities exist. The most common pathogens are coagulase negative *Staphylococcus* and *Staphylococcus aureus* [3]. Besides the use of antibiotics, sufficient debridement seems to be the most important therapy for osteomyelitis. However, in the presence of necrotic bone, a biofilm can develop and reduce the effectiveness of antibiotic therapy by 10^3^ [4]. Although progress has been made, controlling infection in osteomyelitis still remains difficult. In most cases, a definite treatment can only be achieved by intensive debridement resulting in large critical sized defects or even amputation of the infected extremity [5]. Therefore, the purpose of this study was to further investigate in this field and prevent extensive bone loss. Different studies discussed the influence of several factors like TNF-alpha, RANKL and IL-1, which are able to induce bone resorption especially mediated by *S*. *aureus* [6–10]. Moreover, it could be shown that osteogenesis is decreased in inflammatory conditions [11–13]. Most existing animal models for osteomyelitis focus on the phase of infection or developing infection [14–23]. Many of them elucidate the period during and after different therapeutical strategies of osteomyelitic bone [24–35]. However, to our knowledge none of them explores bone healing when the causing agent, i.e. *S*.*aureus*, is removed. The purpose of this study was to establish a new mouse model for further investigations in osteomyelitis treatment. We wanted to translate clinical approaches into a mouse model, which offers a fantastic tool to utilize the entire biomedical armamentarium for in depth analysis. Therefore C57BL6 mice tibias were inoculated with *S*. *aureus* for 2 weeks, followed by thorough debridement and subsequent antibiotic therapy or antibiotic treatment alone respectively. In order to come closest to clinical situation, Gentamicin was used for antibiotic therapy, as it is often used for treatment of osteomyelitis [36]. Moreover adverse effects of Gentamicin on bone regeneration were described in vitro [37, 38]. These effects are taken into account by the use of this antibiotic. The incubation time after bacterial inoculation was set at two weeks because of own experiences and existing animal models [18].

## Materials and Methods

### Mouse Osteomyelitis Model

All experiments were performed in adherence to the National Institute of Health guidelines for the use of experimental animals and after approval by the German legislation.

The protocol was approved by the LANUV (NRW, Germany) (PermitNumber: 84–02.04.2014.A044). Animals were housed and caged individually with free access to water and food under specific pathogen free conditions. All efforts were made to minimize suffering.

60 C57BL6 male and female mice, purchased from Charles River Laboratories (Sulzfeld, Germany) were used. Animals were anesthetized by Ketamin/Xylazin/Acepromazin injection in appropriate doses (Ketamin 100 mg/kgBW, Xylazin 20 mg/kgBW, Acepromazin 3 mg/kgBW). Thereafter, the right leg was disinfected with povidone iodine. A skin incision of 1 cm was made over the proximal medial tibia and the bone was exposed by sharp preparation of the tibialis anterior muscle. Then a hole (1 mm diameter) was drilled into the proximal medial tibia with a brushless micromotor (Ultimate NSK 450), without hurting the contralateral side of the cortex, in order to create an unicortical defect, similar as previously described [39, 40]. Then 0.5 μl of PBS, containing 2000 bacteria, was injected into the medullary cavity of the tibia with a hamilton syringe, whereupon the bone defect was closed with non-resorbable bone wax. For inoculation of bacteria, *Staphylococcus aureus* Rosenbach 1884 was used, which was tested sensitive to gentamicin (for complete resistogram, please see S1 Table in the supporting information). Then the muscle was sutured and the wound closed. Animals were placed on a heating pad and monitored until recovery. Criteria for animals recovered from anesthesia were spontaneous forelimb moving and drinking of water. Animals were monitored tightly after surgery.

**Fig 1:**
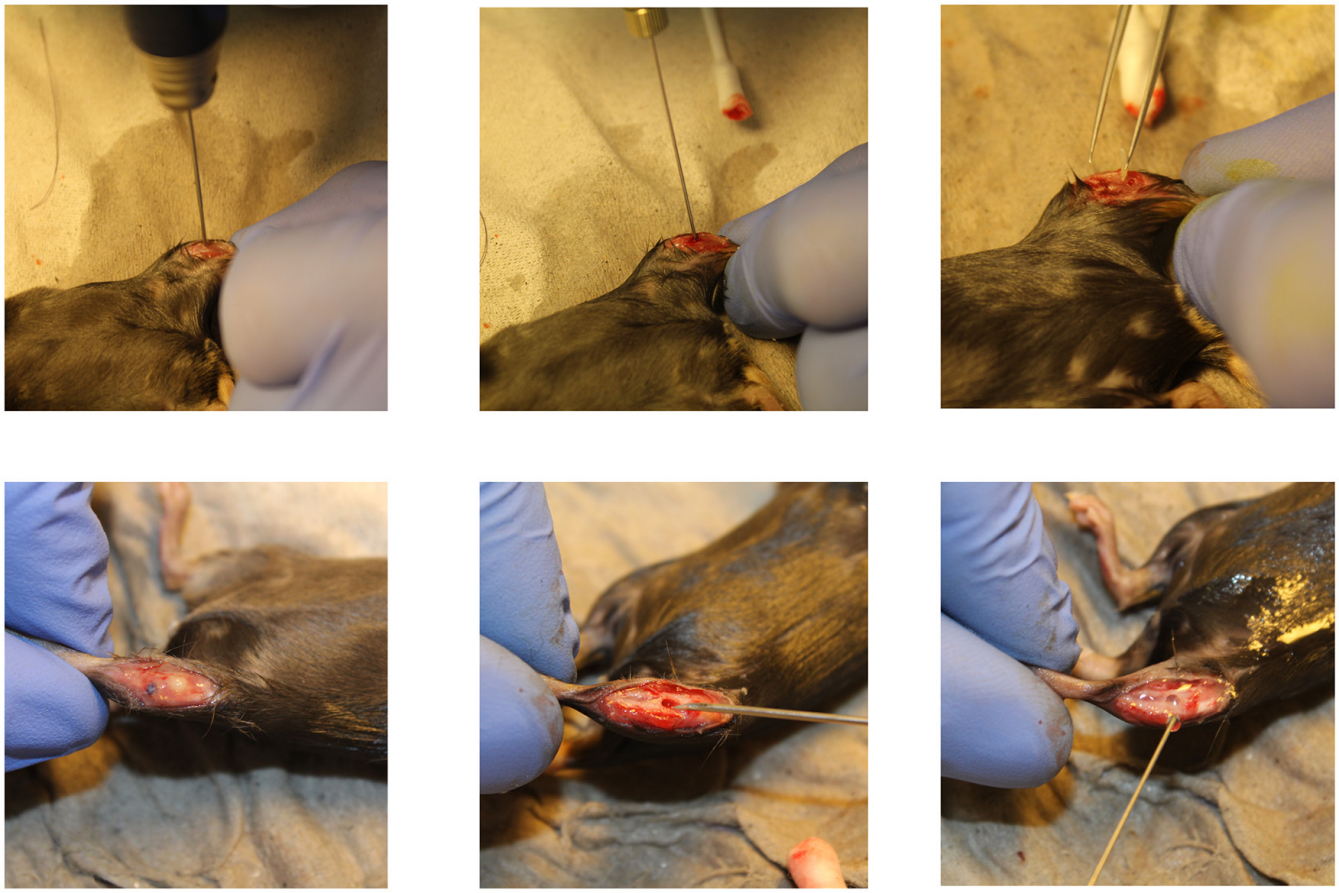
Sequence of surgical procedures. Upper row from left to right: 1. Drilling a 1 mm hole into proximal mouse tibia; 2. Inoculation of bacteria with a hamilton syringe; 3. Sealing of the defect with bone wax. Lower row from left to right: 1. Exposition of infected bone; 2. Debriding infected bone tissue with injection needle; 3. Thorough flushing of debrided bone.

After 2 weeks, animals were prepared for the second surgery as mentioned above. After anesthesia and disinfection of the mouse, a skin incision was made once again over the proximal medial tibia and the bone defect was exposed. Then, infected bone tissue was debrided by the use of a 20 gauge injection needle. The criterion for sufficient debridement was to remove all squashy, necrotic bone material till solely intact bone was left. In addition, edges of the bony defect were sharply scraped with the injection needle. In order to guarantee standardization of this procedure, a custom-made measurement device was used to control defect size after debridement. A detailed sequence of different surgical steps is depicted in Fig 1. Thereafter, debrided defects were rinsed with NaCl. After debridement, the wound was closed and animals were monitored until recovery from anesthesia. For postoperative care, animals received Gentamicin (52 mg/kg per day) subcutaneously. After 1 (debrided week 1; (deb.W1)) or 2 (deb.W2) weeks, mice were sacrificed and the tibia was removed for further analysis. One control group, consisting of animals that underwent surgery in identical steps as described above, but without inoculation of bacteria, was included into the study. After debridement, these animals also received antibiotic treatment for 1 (debrided no infection; (debnoi.W1)) or 2 (debnoi.W2) weeks. In addition, some animals received sole antibiotic treatment for 1 (nondebrided; (nondeb.W1)) or 2 weeks (nondeb.W2), therefore omitting the surgical procedure of debriding infected bone. Groups consisted of at least 5 animals.

### Microbiotic Assessment

In order to prove the efficacy of surgical debridement, smears from the infected bone of all animals were taken intramedullary right before debridement and 1 or 2 weeks after surgical debridement and antibiotic therapy. The smears were inoculated on CHROMagar plates (BBL CHROMagar Staph aureus, BD Biosciences, Heidelberg, Germany) and incubated under aerobic conditions for 48 hours at 37°C after which *S*. *aureus* could be identified by bright violet colonies on the plate.

### Histology and GRAM staining

For histology, tibiae were taken and fixed in 4% paraformaldehyde overnight, decalcified in 19% EDTA and paraffin embedded. Tibiae were then longitudinally sectioned at 9 μm on a microtome. After sectioning, GRAM staining was performed. Briefly, slides were first incubated with a few drops of carbol gentianaviolet for 2 minutes and rinsed with Lugol solution. Thereafter, slides were incubated with Lugol solution for 2 minutes and rinsed with distilled water. Then slides were decolored with GRAM’s decolorizing solution and rinsed with distilled water. A few drops of carbolic fuchsine solution were added and incubated for 30 seconds. After carefully rinse with distilled water, slides were dehydrated by ascending alcohol solution and cleared with xylene before mounting with Roti-Histokit (Carl Roth, Karlsruhe, Germany). In order to detect remaining bacteria within the bony defect, the entire defect site was examined at 100x magnification and characteristic photographs were taken, using a Zeiss Axioplan microscope. In addition, GRAM stainings of infected animals before debridement were performed, in order to characterize infection status.

For examination of new bone formation, aniline blue staining was performed after a standard protocol. The stained sections were then photographed with Leica digital imaging system at 5x magnification and evaluated with Photoshop. The aniline blue-positive pixels were semi-automatically selected with Magic Wand tool (tolerance: 60, noncontiguous). Cortical surface and bone chips were manually deselected. The average of positive pixels for each group was taken and Student’s t test was used for statistical analyses, a p-value p<0.05 was considered statistically significant.

### DNA-extraction

For DNA-Extraction AllPrep^®^ DNA/RNA/Protein Mini Kit (Qiagen, Hilden, Germany) was used after manufacturer’s instructions. Extracted DNA was stored at -20°C.

In order to quantify the amount and purity of isolated DNA, OD 260/280 ratio and total DNA amount was measured photometrically.

### RT-qPCR

For identification of *S*. *aureus* within the isolated DNA specimens, a TaqMan assay purchased by Life Technologies (Life Technologies, Carlsbad, USA) was used. TaqMan primers and probes were already established by a study analyzing *S*. *aureus* in contaminated food [41]. For identification of *S*. *aureus*, the htrA gene was targeted.

As a reference, infected bones of four animals were harvested and DNA was isolated. The amount of S. aureus-DNA was defined as 100%, shown at day 0.

Quantitative real time PCR reactions were performed in a volume of 15 μl, containing 7.5 μl of TaqMan Environmental Master Mix 2.0, 2 μl of template genomic DNA (20 ng), 0.75 μl of TaqMan assay and 4.25 μl of molecular grade water. The reaction was carried out in 96 well plates. Each sample was tested in triplets. To evaluate background signal, no-template controls were assayed. As a positive control, gDNA extracted from cultured *S*. *aureus* was utilized.

For calculation of the copy number of *S*. *aureus*, a calibration line using 10^3^ to 10^7^ copies of the *S*. *aureus* genome was established.

The cycling conditions were adjusted to initial denaturation of 2 minutes at 50°C and 10 minutes at 95°C followed by 40 PCR cycles at 95°C for 15 seconds and 60°C for 1 minute, using 7500 Real Time PCR System (Applied Biosystems, Darmstadt, Germany).

### Statistics

P-values were calculated by Student’s t-test when comparing two groups and one way ANOVA when comparing more than two groups. For post-hoc comparisons Tukey’s test was used. Statistical significances were set at a p-value < 0.05.

## Results

### Surgical procedure

All animals (60/60) survived the surgical procedures. Animals were observed twice a day postoperatively. One animal had to be sacrificed after surgery because of a painful leg fracture and was not included into the study. No postoperative skin infection or fever could be observed.

### Microbiotic Assessment

**Fig 2:**
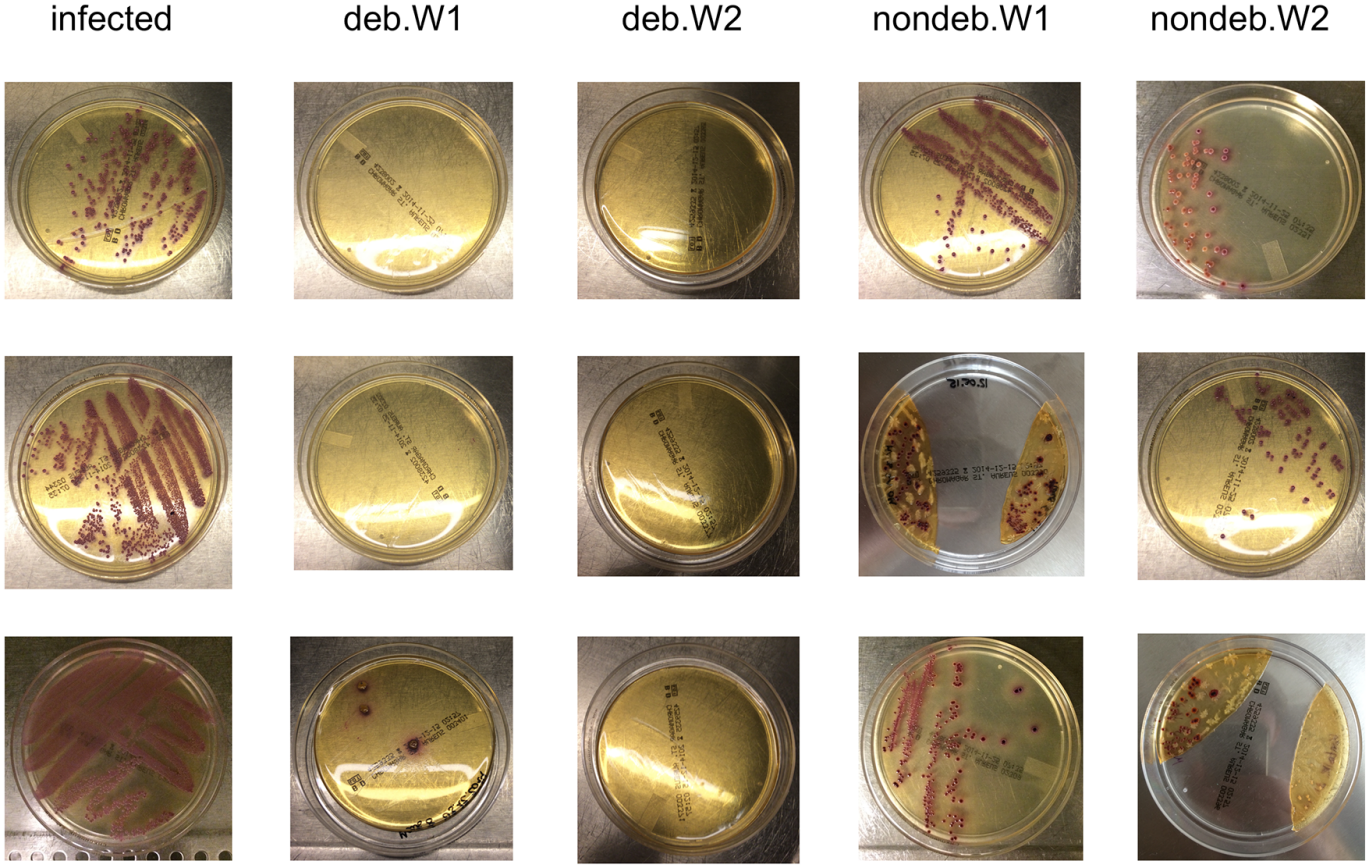
Bone smears on culture plates as infection control. Culture plates (CHROMagar Staph aureus, BD, Franklin (USA)) with smears of infected bone before debridement and smears taken from animals of deb.W1 and 2 and nondeb.W1 and 2.

All smears taken before debridement showed characteristic accretion of *S*. *aureus*, depicted in Fig 2. One smear of deb.W1 showed few characteristic CFUs on the agar plate. One smear of deb.W2 showed one CFU but no characteristic purple colony of *S*. *aureus*. All other smears of debrided animals were sterile and showed no accretion of any germ (Fig 2). In contrast, smears taken from nondebrided animals of nondeb.W1 and 2 showed characteristic growth of CFUs on agar plates. Of note, no CFUs were detected in debrided, non-infected controls (debnoi.W1 + debnoi.W2).

### Histology

**Fig 3:**
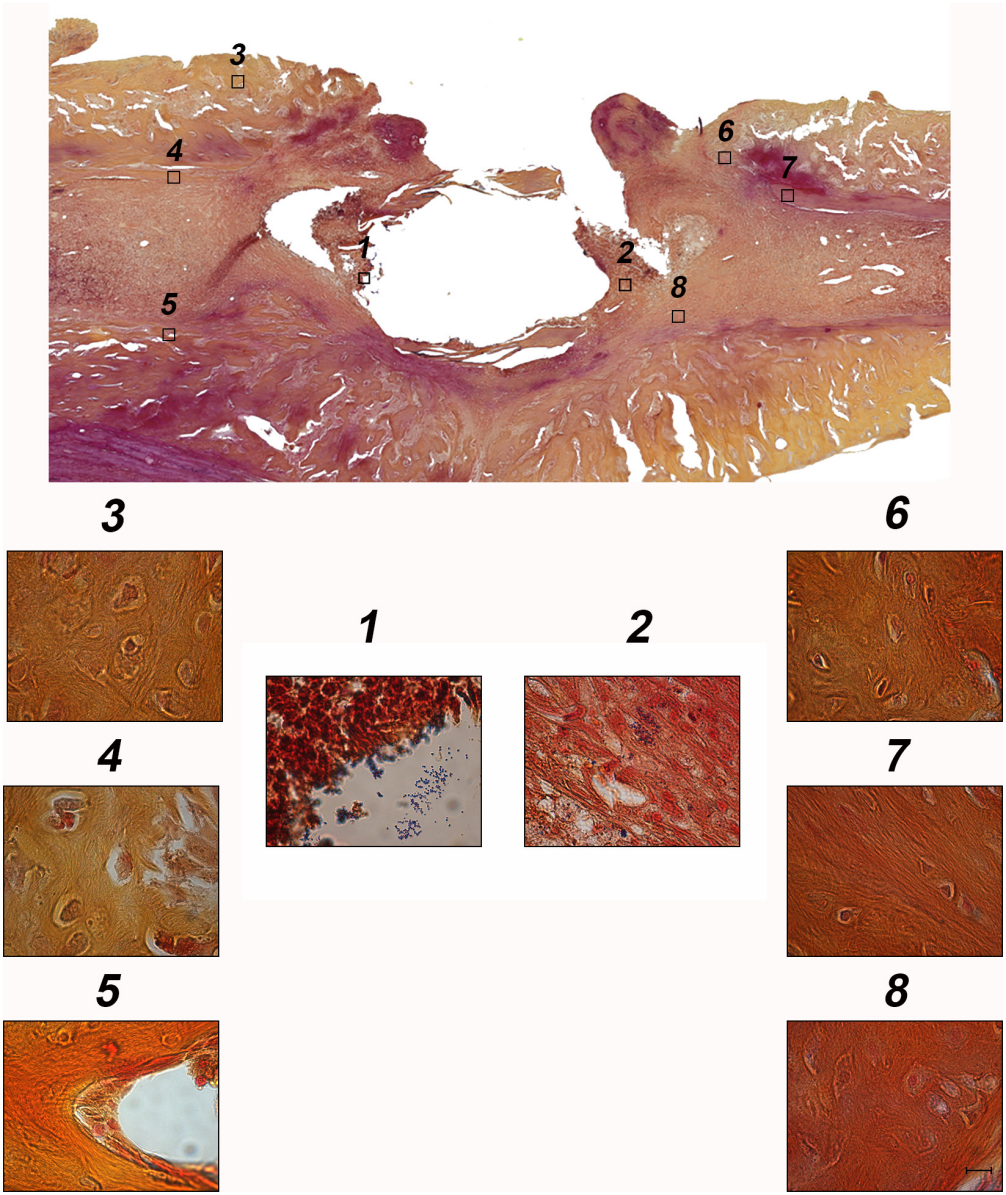
GRAM staining of infected tibiae. Bacteria could only be observed within the defect site, whereas surrounding tissue showed complete absence of GRAM-positive bacteria. Numbers in overview above correspond with insets, which are enlarged below. Scale bar represents 10 μm.

**Fig 4:**
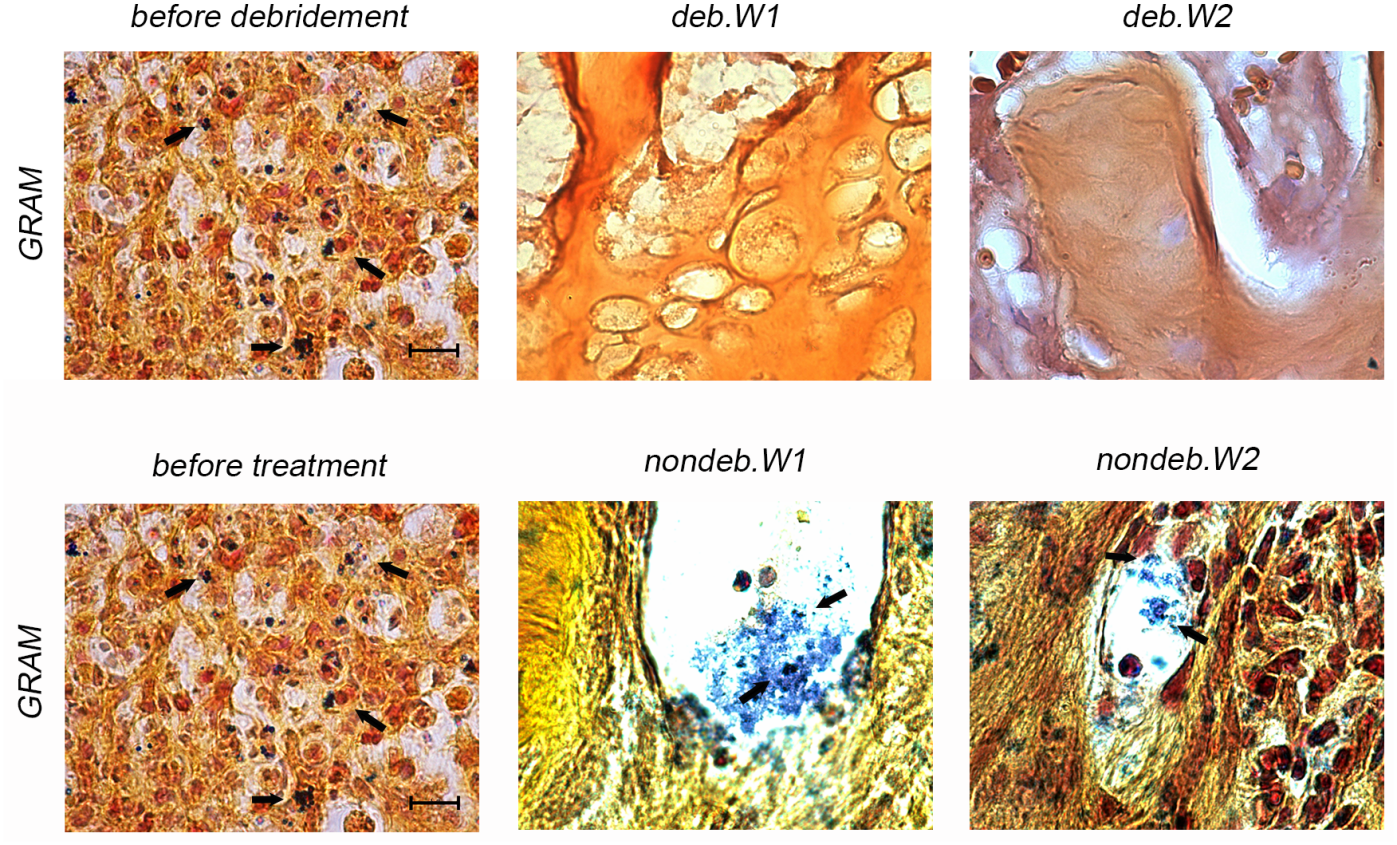
GRAM staining of defects of deb.W1 and 2 and nondeb.W1 and 2. Whole defect was examined and characteristic photographs were taken in order to detect *S*. *aureus*. In deb.W1, *S*. *aureus* could be detected in two animals sporadically. No bacteria at all could be descried after 2 weeks of antibiotic treatment in deb.W2. Characteristic clusters of *S*. *aureus* could be detected in all animals of nondeb.W1 and 2. Scale bar represents 10 μm.

GRAM-staining of infected animals revealed the presence of bacteria within the defect site but not in the surrounding tissue (Fig 3). Looking through GRAM staining of deb.W1 and 2, hardly any *S*. *aureus* bacteria could be detected. In deb.W2, no bacteria could be found at all, in deb.W1, two animals showed sporadic appearance of bacteria within the entire defect site (Fig 4). GRAM staining of nondeb.W1 and 2 revealed appearance of multiple GRAM-positive clusters within the whole defect site.

**Fig 5:**
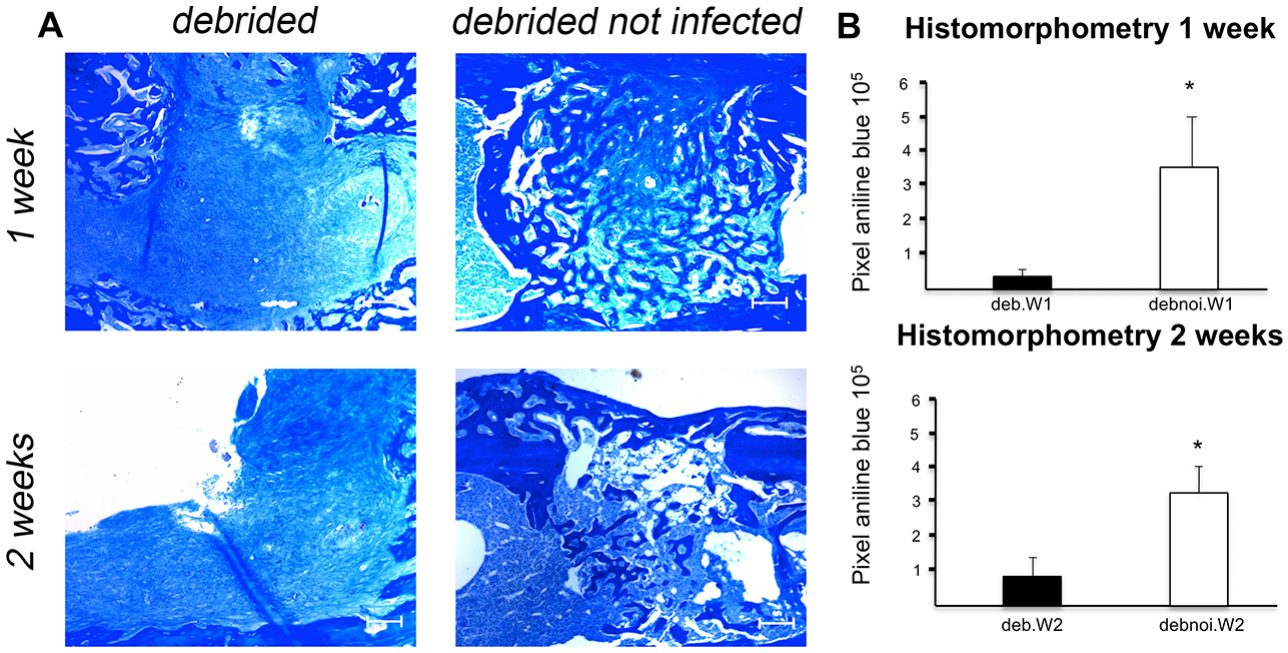
A. **Aniline blue staining of deb.W1 and 2 with corresponding controls**. In the upper row staining of osteomyelitic animals after 1 and 2 weeks can be seen, whereas staining of noninfected control animals are presented in the lower row. After 1 week of antibiotic treatment newly formed bone is significantly increased within control group compared to osteomyelitic animals. Scale bar represents 200 μm. B. Histomorphometry of aniline blue staining of deb.W1 and 2. After 1 week (deb.W1) and 2 weeks (deb.W2) of antibiotic treatment, debrided, previously infected animals show less new bone formation than controls.

In order to assess new bone formation, aniline blue staining was performed, and animals of deb.W1 and 2 were compared with animals of control group 1 and 2, as shown in Fig 5. Bone healing of osteomyelitic animals undergoing debridement (deb.W1 and 2) was significantly decreased in comparison to control animals (debnoi.W1 and debnoi.W2).

Histomorphometrical analyses of aniline blue stainings were performed to quantify osteogenesis of debrided animals and controls. The amount of newly formed bone was significantly decreased in the deb.W1 and W2 groups as compared to debnoi.W1 and W2 controls. Of note, bone formation in animals of nondeb.W1 and.W2 was similar to deb.W1 and W2.

### RT-qPCR

**Fig 6:**
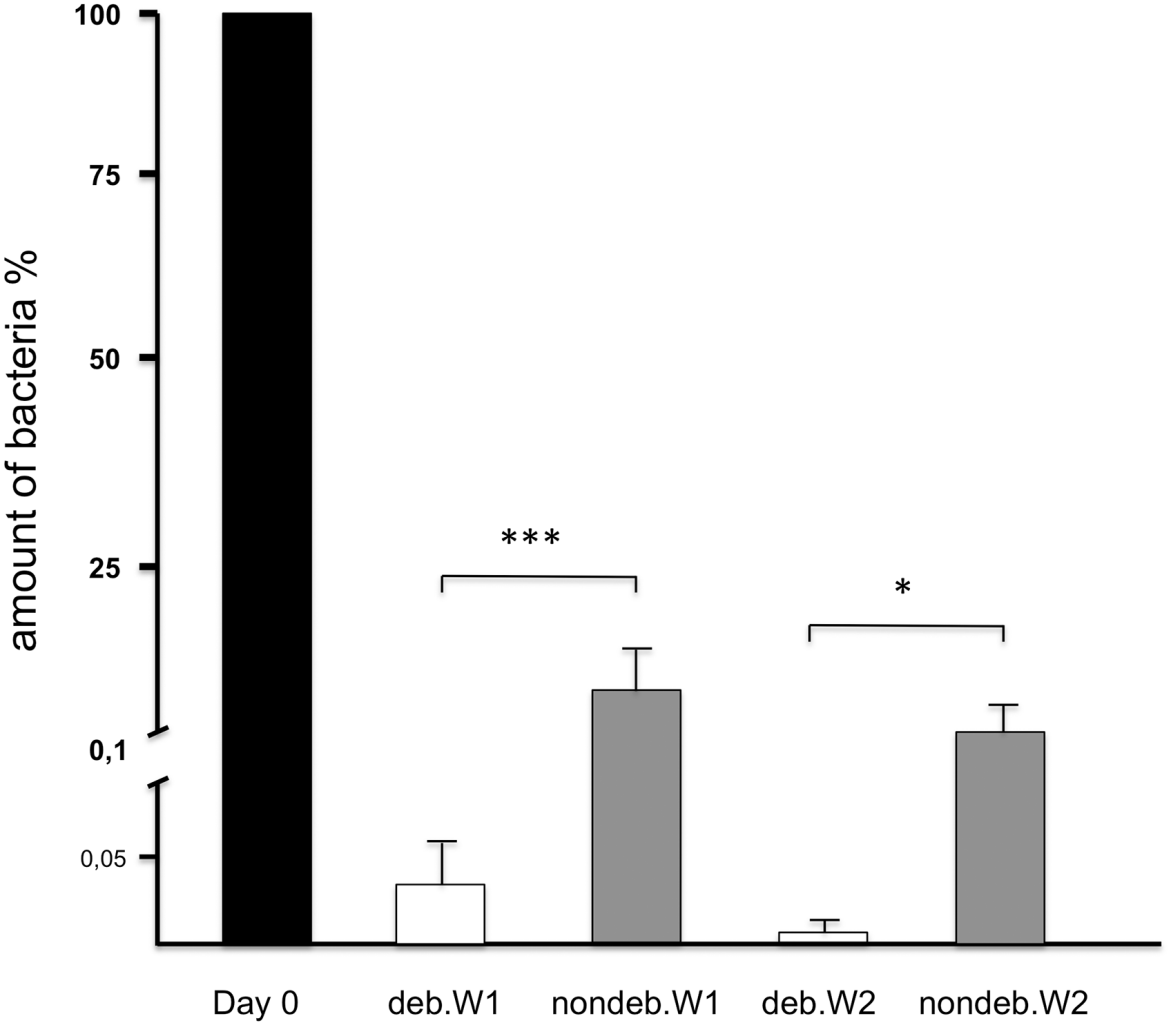
Results of qRT-PCR detecting Staph aureus DNA. The amount of *S*. *aureus* within the infected bone of deb.W1 and 2 and nondeb.W1 and 2 in comparison to initial amount of bacteria before debridement (Day 0) is depicted. Significant differences between deb. and nondeb.W1 and 2 could be found.

A fundamental reduction of the bacterial burden after debridement and antibiotics was shown via RT-qPCR. In deb.W1 only a diminutive amount of bacterial DNA was detectable, whereas in deb.W2 almost zero *S*. *aureus* gDNA was detected in most of the cases, as shown in Fig 6. Furthermore, debrided (deb.W1 and 2) animals showed significantly lower amount of bacteria via debridement, compared to nondebrided (nondeb.W1 and 2) animals, exclusively treated with antibiotics.

## Discussion

In the current study, we were able to develop a mouse model for studies related to improve treatment of posttraumatic osteomyelitis. Our results exhibit that, after *S*. *aureus* bacterial inoculation and incubation, a steady infection in tibia was generated. According to the classification of Cierny and Mader, a localized type of osteomyelitis was created in hosts with uncompromised immune systems, corresponding to type III A osteomyelitis [42], which was verified by GRAM staining of infected mouse tibiae. After debridement and antibiotic therapy, bacterial count was radically reduced or eradicated. This was verified by three different detection methods: CHROMagar plates, GRAM staining and RT-qPCR.

A number of animal models were created, in order to study the effects of bacterial infections on bone healing and to find new strategies for treatment. *S*. *aureus* strain for bacterial inoculation was used in almost all of them, since this is the most common pathogen in the setting of osteomyelitis [3]. In most animal models, implant associated infections were examined or fracture models that needed to be stabilized by foreign bodies [20–22, 30, 32–34, 43–49]. Furthermore, rats and rabbits were used in most studies, as they are big enough to create stable long bone infections without tremendous efforts, but on the other hand are small enough to be housed and handled easily [22, 43–45, 48–51]. Moreover, different ways of bacterial application were chosen. For example, Speers *et al*. created a stable osteomyelitis in chicken, by applying *S*. *aureus* via the wing vein [52]. Most investigators however, applied *S*. *aureus* directly to the bone. In recent years, many animal models, dealing with different therapeutic options for osteomyelitis, were published [24–35]. For instance, Brin et al. studied the effects of locally applied Gentamicin in an intramedullary osteomyelitis model [24]. Furthermore, Inzana et al. compared the efficacy of different antibiotic-laden spacers, diminishing bacterial burden, in a mouse femur osteomyelitis model [34]. It is well known that presence of bacteria within the infected bone leads to osteolysis and diminished bone healing, even after surgical debridement [20, 21, 34], but it remained unclear if bone regeneration is still diminished after infection is treated, by eradicating the causing microorganisms. Therefore, we created this mouse model to analyze bone regeneration in this particular condition and establish a platform for further analysis. In this context, our study revealed diminished bone healing after the absence of S. aureus (Fig 5). Moreover, the resulting bony defect in animals with impaired bone healing after infection and debridement can be used for further experiments, improving bone regeneration.

Effectiveness of treatment was verified with different methods, similar to clinical approaches. Microbiological assessment via CHROMagar plate is a commercially available and approved assessment for testing specimens on *S*. *aureus* colonization [53]. Results of smears, taken from bone material, showed a bacterial colonization of one specimen one week after antibiotic treatment, whereas in deb.W1 no bacterial colonization characteristic for *S*. *aureus* was detected. These observations correspond with results of GRAM staining. While specimens of deb.W2 seemed to be sterile, only two specimens of deb.W1 showed sporadic bacterial colonization. The results obtained with the RT-qPCR, which can be utilized to detect even single copies of bacterial DNA, showed similar results: in deb.W1 single bacteria could be detected, whereas in deb.W2 hardly any *S*. *aureus* DNA could be detected. The results of antibiotic assessment and GRAM staining showed effective treatment of infected bones after 1 and 2 weeks. Debridement and antibiotic treatment for one week seemed to be effective to remediate the infected bone. Further treatment with antibiotics for 1 more week seemed to have only little additional effect on bacterial colonization at the defect site, validated by comparing group 1 and 2.

Additionally, the role of surgical treatment was examined via antibiotic treatment of infected animals without debridement. All three detection methods showed residual infected bone within these animals, emphasizing the importance of surgical debridement. Interestingly, new bone formation was significantly decreased in all osteomyelitic groups, compared to controls. Debrided bone after infection seemed to have limited osteogenic potential, even after absence of *S*. *aureus*.

Our new murine model of tibial osteomyelitis has a lot of advantages as it needs no internal or external stabilization by implants and is easy and reproducible. Furthermore, the use of mice provides numerous advantages. Mice are extremely useful for understanding the pathophysiology of osteomyelitis, as abundant genetically modified mice are commercially available and the entire armamentarium of biomedical tools is available. Although results show a reliable reduction of bacteria after bone infection, sporadic presence of bacteria could be detected in all three assays. This circumstance displays clinical situations, in which remediation of osteomyelitis can not be granted in all cases. In order to control sufficient surgical therapy of bone infection for subsequent studies, we recommend the use of a microbiotic assessment, i.e. CHROMagar plates, to verify absence of bacteria before further tests.

Taking these results together, we could create a stable and reproducible infectious situation in murine tibia, which could be treated effectively by debridement and antibiotics subsequently. The effectiveness of treatment was validated by three different methods. Thus, we were able to develop a translational murine model for osteomyelitis, mimicking the situation in septic surgery.

## Supporting Information

S1 ARRIVE Checklist Within the experiments of this paper all ARRIVE-guidelines were obeyed. (PDF)

S1 Table Resistogram of utilized *S*. *aureus*. The used germ is sensitive to most antibiotics including gentamicin. (Abbreviations: R = resistant; S = sensitive).
(TIF)
