# Supplementary figures and images for: Surgical Debridement Is Superior to Sole Antibiotic Therapy in a Novel Murine Posttraumatic Osteomyelitis Model
Source: PLoS One. 2016 Feb 12;11(2):e0149389. doi: 10.1371/journal.pone.0149389 (PMC4752466; doi:10.1371/journal.pone.0149389)

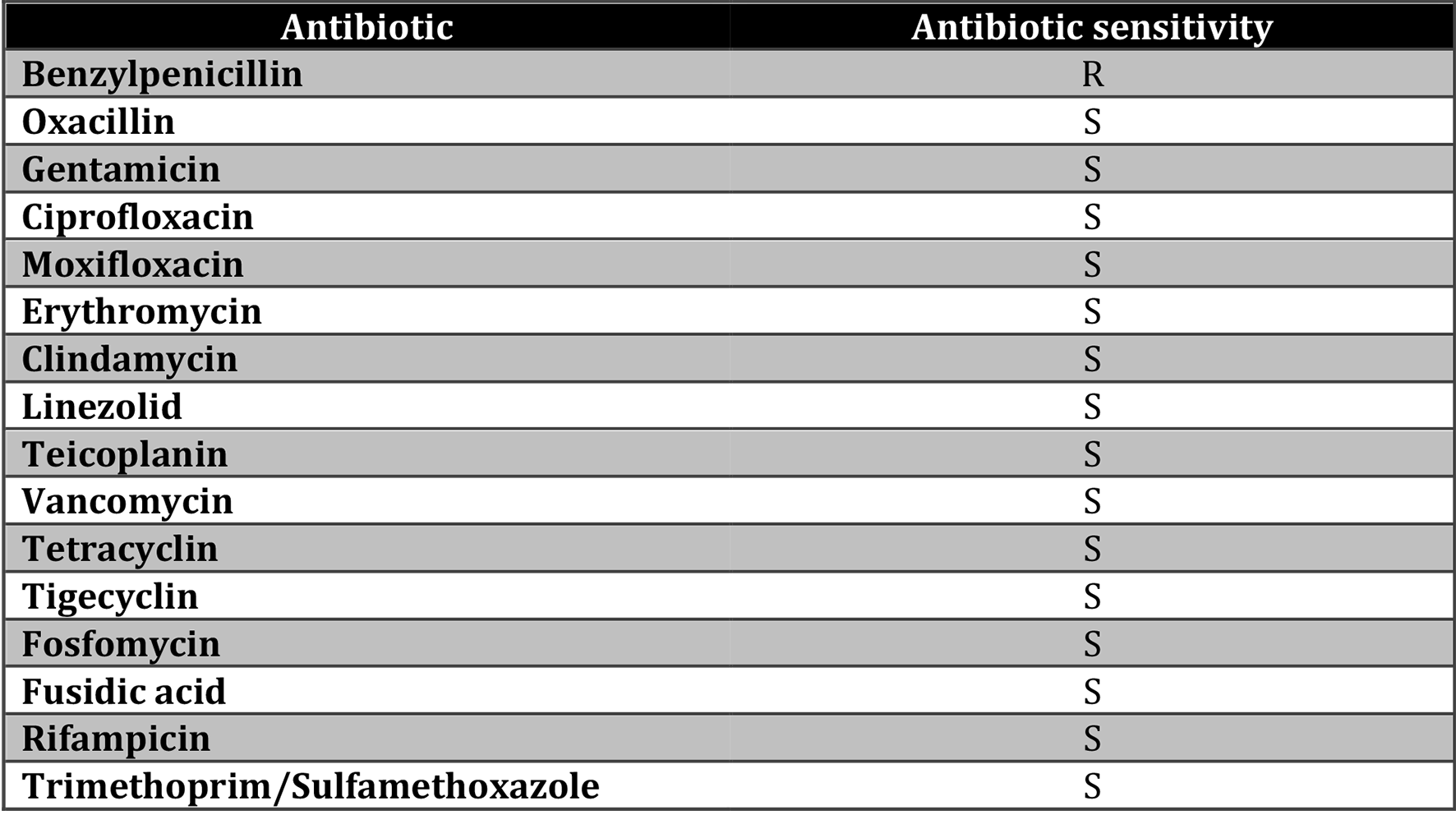

Supplement: S1 Table — The used germ is sensitive to most antibiotics including gentamicin. (Abbreviations: R = resistant; S = sensitive). (TIF) [file pone.0149389.s002.tif]
